# Synthesis and Biodistribution of ^99m^Tc-Labeled PLGA Nanoparticles by Microfluidic Technique

**DOI:** 10.3390/pharmaceutics13111769

**Published:** 2021-10-22

**Authors:** Michela Varani, Giuseppe Campagna, Valeria Bentivoglio, Matteo Serafinelli, Maria Luisa Martini, Filippo Galli, Alberto Signore

**Affiliations:** Nuclear Medicine Unit, Department of Medical-Surgical Sciences and of Translational Medicine, Faculty of Medicine and Psychology, “Sapienza” University of Rome, 00161 Rome, Italy; gius.campagna@gmail.com (G.C.); valeria.benti@gmail.com (V.B.); matteo.serafinelli@gmail.com (M.S.); luisetta84m@libero.it (M.L.M.); filippo.galli@uniroma1.it (F.G.); alberto.signore@uniroma1.it (A.S.)

**Keywords:** radiolabeled nanoparticles, poly (lactic-co-glycolic acid) (PLGA), nuclear medicine, microfluidics

## Abstract

The aim of present study was to develop radiolabeled NPs to overcome the limitations of fluorescence with theranostic potential. Synthesis of PLGA-NPs loaded with technetium-99m was based on a Dean-Vortex-Bifurcation Mixer (DVBM) using an innovative microfluidic technique with high batch-to-batch reproducibility and tailored-made size of NPs. Eighteen different formulations were tested and characterized for particle size, zeta potential, polydispersity index, labeling efficiency, and in vitro stability. Overall, physical characterization by dynamic light scattering (DLS) showed an increase in particle size after radiolabeling probably due to the incorporation of the isotope into the PLGA-NPs shell. NPs of 60 nm (obtained by 5:1 PVA:PLGA ratio and 15 mL/min TFR with ^99m^Tc included in PVA) had high labeling efficiency (94.20 ± 5.83%) and >80% stability after 24 h and showed optimal biodistribution in BALB/c mice. In conclusion, we confirmed the possibility of radiolabeling NPs with ^99m^Tc using the microfluidics and provide best formulation for tumor targeting studies.

## 1. Introduction

The design of targeted drug delivery systems using nanomaterials has rapidly spread in medicine, due to the possibility of improving the targeting and release of drugs from these nano-systems, avoiding their premature biodegradation, off-target and systemic toxicity [1]. The ideal delivery system should satisfy several requirements like biodegradability, biocompatibility, non-immunogenicity and non-toxicity in a biological system. Additionally, they should have the capability of a high load of drugs and administer them to the target with controlled release and distribution, with minimal losses and a prolonged release in the desired site [2,3]. Nanomaterials match these characteristics as they can be used to fabricate several types of nanoparticles (NPs), formulated with organic, inorganic, or hybrid core. The physico-chemical parameters of NPs such as size, shape, surface charge, and materials of the structure, are fundamental for their biodistribution, excretion, pharmacokinetics, targeting, and therefore therapeutic efficacy [4,5]. Polymeric NPs are composed of biodegradable and biocompatible polymers, are easily synthetized and can encapsulate or absorb surface insoluble molecules, therefore they have been extensively studied as vehicles for controlled drug delivery [6,7]. The polylactide-co-glycolic acid (PLGA) are polymeric NPs already approved by Food and Drug Administration (FDA) and European Medicine Agency (EMA) to deliver therapeutic agents parentally administered [8]. PLGA-NPs are naturally degraded in the body by hydrolysis of ester bonds between the lactic (PLA) and glycolic acid (PGA) monomers, metabolized by the Krebs cycle, and excreted by the lungs as carbon dioxide and water or by urine in a non-toxic way [9,10]. The retention of PLGA-NPs at the target site and also the effect of the entrapped drug depends on the composition of polymers. The PLA:PGA ratio influences the degradation kinetics of NPs since a high PLA monomer ratio causes hydrophobicity of PLGA-NPs and their degradation occurs more slowly [11]. PLGA with a ratio of 50:50 is the most used copolymer in nanomedicine, due to its fastest degradation rate and therefore faster drug release rate than other formulations [12]. Drug release can be achieved by passive diffusion from polymer barrier or by erosion of the PLGA-NPs structure [13]. When PLGA-NPs are synthetized, it is important to consider the physical and chemical properties of polymers, and also the complexity of the process to achieve the optimal drug delivery system [14]. In recent years, an innovative synthesis method has been explored, called ‘microfluidic technique’, that overcomes some limitations given by the bulk nanoprecipitation method [15,16]. In this method, an aqueous solution has been mixed with a solvent phase (as acetonitrile, acetone, or ethanol) and this causes the precipitation and nucleation of polymers, assembling in NPs [17]. The bulk-synthesis technique has been extensively studied for NPs production, but suffers from poor batch-to-batch reproducibility, long preparation times, and several steps of manipulation that can lead to NPs aggregation [18,19]. The microfluidic method offers the potential of a controlled system in which the organic and the aqueous phases are mixed in a microfluidic chip with precision settings, such as the total flow ratio (TFR) and the flow rate ratio (FRR) [20]. The TFR is the total speed in mL/min at which both the fluids are mixed in the microfluidic platform, and the FRR is the volumetric ratio of the mixed organic and aqueous phases. This leads to precise NPs size control and a high degree of particle uniformity (polydispersity index below 0.2), allowing batch-to-batch reproducibility. These are crucial factors for GMP production of radiolabeled PLGA-NPs to be used in clinical practice. In our study, we explored the possibility to produce radiolabeled PLGA-NPs with a novel microfluidic system based on a Dean-Vortex-Bifurcation Mixer (DVBM) [21]. Despite the wide use of PLGA-NPs as delivery drug system, the knowledge of a reproducible incorporation of imaging agents into PLGA-NPs is yet to be explored. In our previous study, we demonstrated how fluorescent PLGA-NPs were able to passively accumulate in a syngeneic tumor model of sarcoma, with maximum uptake at 72 h [22]. Then, the possibility to combine diagnostic agents with therapeutics molecules in NPs, makes them a promising theranostic tool. The aim of this study was to investigate a novel microfluidic process for manufacturing PLGA-NPs loaded with diagnostic isotope, as a necessary step for future theranostic application. The radioactive isotope allows a signal detection from deeper tissues, overcoming the limitation of fluorescence and enabling a translational potential of NPs as imaging probe. Therefore, formulation parameters (TFR and FRR) were set to achieve the best encapsulation efficiency of technetium-99m (^99m^Tc). 

## 2. Materials and Methods

### 2.1. Materials

Poly (lactic-co-glycolic acid) (lactide to glycolide ratio 50:50, acid endcap, Mw: 35.000–45.000 Da) was purchased by Akina, Inc. (West Lafayette, IN, USA). Polyvinyl alcohol (PVA Mw: 31,000 Da). Acetonitrile (ACN) (HPLC grade) and ultrapure water (HPLC grade) were supplied by Merck (Darmstadt, Germany). Technetium-99m was obtained by elution from ^99^Mo/^99m^Tc generator.

### 2.2. Synthesis of PLGA-NPs

An innovative microfluidic mixer (NanoAssemblr^®^ Benchtop, Precision NanoSystems Inc., Vancouver, BC, Canada) was used to manufacture PLGA-NPs. The platform uses compatible syringes to inject solutions in two different channels on a chip incorporated in a disposable cartridge inserted into a microfluidic device controlled by a laptop. The chip is composed by a plurality of mixers, defined as Dean-Vortex-Bifurcation Mixer (DVBM). Each DVBM is characterized by a bifurcation with four toroidal elements arranged in series to mix the liquid provided [23]. Each DVBM in turn contains a plurality of toroidal mixing elements. The fluids under a constant pressure are pumped into the mixer and flow through curved channels. The increased speed of the flow in the center of the channel causes it to deflect outward due to the higher centripetal force. This flow is opposed to another inward flow at the top and the bottom of the channel, creating a pair of counter-rotating vortices, known as Dean vortex. DVBM has a fully planar geometry which reduces the possibility of material clogging or adsorption and also allows for higher flow rates on larger devices. The non-turbulent process condition ensures reproducible results with high NPs quality. 

To synthetize PLGA-NPs, the polymeric materials (PLGA 50:50) were dissolved in acetonitrile (ACN) at a concentration of 5 mg/mL. PVA (2%, *w*/*v*), dissolved in ultrapure water, was used as aqueous phase. The solutions were loaded in two different and disposable syringes and injected into a separate inlet point on the mixer. 

In order to evaluate the effect of the different instrument parameters, TFR 8, 12, and 15 mL/min and FRR 1:1, 3:1, and 5:1, were tested. Nine parallel batches were synthetized and analyzed (Table 1).

### 2.3. Synthesis of ^99m^Tc-PLGA-NPs

In order to find the best radiolabeling approach, two different approaches were tested for the microfluidic syntheses of ^99m^Tc-radiolabeled PLGA-NPs (Figure S1). 

In the first one, 100 µL of ^99m^TcO_4_^−^ (NaCl) were added dropwise to 1 mL of PLGA polymers (5 mg/mL ACN) and injected into the organic inlet of the microfluidic mixer. PVA (2%, *w*/*v*) dissolved in ultrapure water was injected through the other inlet of the microfluidic mixer. We called these batches ^99m^Tc-polymer-(PLGA-NPs).

In the second one, 100 µL of ^99m^TcO_4_^−^ (NaCl) were added dropwise to 2 mL of the aqueous phase (PVA, 2%) and injected into the appropriate inlet of the microfluidic mixer. PLGA polymers (5 mg/mL ACN) were injected into the other inlet. We called these formulations ^99m^Tc-PVA-(PLGA-NPs).

For both the radiolabeling approaches, the different instrument parameters described in Table 1 were tested, thus obtaining a total of 18 different formulations.

To radiolabel each batch with the same activity of ^99m^TcO_4_^−^ (5 mCi), we used different starting concentrations of isotope, considering the different FFR, as shown in Table 2.

### 2.4. Quality Controls

#### 2.4.1. Particle Size Distribution and Zeta Potential (ζ) Measurements

Volume-average diameter (nm) and polydispersity index (PDI) of NPs were analyzed by dynamic light scattering (DLS) using a NanoZetaSizer analyzer (Malvern Instruments Ltd., Malvern, UK). After the batch synthesis with NanoAssemblr^®^, native PLGA-NPs, ^99m^Tc-polymer-(PLGA-NPs), and ^99m^Tc-PVA-(PLGA-NPs) were loaded into the instrument and analyzed. All the batches were analyzed pre- and post-PD-10 purification step.

Briefly, 10 μL of each sample were suspended with 90 μL of ultrapure water and loaded in Sarstedt polystyrol/polystyrene cuvettes (10 × 10 × 45 mm) for the measurements performed at 25 °C. To study the surface charge of NPs, the zeta potential analysis was performed with the same instrument. In total, 20 μL of NPs were suspended with 980 μL of ultrapure water, sonicated, and loaded in Malvern folded capillary cells for measurements. All measurements were performed in triplicate and mean values ± standard deviation (SD) are reported.

#### 2.4.2. Labeling Efficiency and Yield Calculation of ^99m^Tc-PLGA-NPs

The ITLC was used to evaluate the presence of unlabeled ^99m^Tc in the preparation (free pertechnetate ^99m^TcO_4_^−^) with sodium chloride (NaCl 0.9%) as mobile phase. The free pertechnetate present in the solution migrates to the front of the strip (Retention Factor = 0.9), while the radiolabeled compound remained at the bottom (Retention Factor = 0.1). ITLC strips were spotted with 2–3 µL of the solution at 1 cm above the bottom, and immediately put into a falcon vial with the mobile phase. After migration of the mobile phase up to 1 cm from the top, the strip was dried and analyzed in a Radio-TLC Imaging Scanner (Bioscan, Inc, Poway, CA, USA). Labeling efficiency was calculated as percentage of NPs activity over total.

After the synthesis, a PD-10 desalting column containing Sephadex G-25 resin (GE Healthcare, Uppsala, Sweden) was used to purify the radiolabeled NPs from the unlabeled technetium present in solution. Briefly, 1 mL of ^99m^Tc-PLGA-NPs was added to column, then 1.5 mL of PBS were added to completely fill the column volume and the sample eluted was discarded. In total, 10 mL of PBS were eluted and collected in 20 fractions (500 µL each). The acetonitrile and the other small contaminants in solution were removed in the same purification step. 

Each fraction was counted in a single-well NaI γ-counter (AtomLab, 500-Biodex) and the yield was calculated as follows:

YIELD (%) = 100 × [mCi_(_^99m^_Tc-PLGA-NPs)_/mCi_(tot)_]

where mCi_(tot)_ is the starting activity used (5 mCi), while mCi_(_^99m^_Tc-PLGA-NPs)_ is the amount of mCi from the first 5 fractions in which the labeled NPs were eluted.

#### 2.4.3. “In Vitro” Release Study

^99m^Tc-polymers (PLGA-NPs) and ^99m^Tc-PVA (PLGA-NPs) synthetized with the formulation parameters of batch #9 were selected for further experimentations. 

In vitro release of technetium-99 from selected radiolabeled NPs was evaluated in NaCl 0.9%. The radiolabeled compound was incubated at room temperature for 1, 3, 6, and 24 h. At the end of each time point, each batch was analyzed by ITLC as previously described. 

Size and PDI of labeled NPs were also measured by DLS over time, being important indicators of NPs aggregation.

### 2.5. In Vivo Biodistribution Studies

All animal experiments were carried out in compliance with the local ethics committee and in agreement with the National rules and the EU regulation (Study 204/2018-PR). Biodistribution of ^99m^Tc-PLGA-NPs was studied in 18 normal 8-week-old female BALB/c purchased from Envigo. Mice were divided in two groups of nine mice each. Group 1 was injected ^99m^Tc-polymers (PLGA-NPs) batch #9 formulation, group 2 was injected ^99m^Tc-PVA (PLGA-NPs) batch #9 formulation. The 100 μCi (100 μL) of radiolabeled nanoparticles were injected into the lateral tail vein of each animal. After each time point (1, 6, 24 h) three mice per group were sacrificed; blood samples and major organs (small and large bowel, kidneys, spleen, stomach, liver, muscle, bone, lungs, heart) were collected and weighted. The radioactivity from each vial was counted in a single-well γ-counter (PerkinElmer, Waltham, MA, USA). The percentage of injected dose per organ (%ID) and percentage of injected dose per gram (%ID/g) were calculated. ^99m^Tc standards were prepared by dilution method and appropriate decay corrections were applied to all the samples.

## 3. Statistical Analysis

Continuous variables are shown as mean ± SD (standard deviation). The differences between native vs. ^99m^Tc-polymers (PLGA-NPs) vs. ^99m^Tc-PVA (PLGA-NPs) of the continuous variables were tested by GLM (General Linear Model) when the normality of the residuals was verified or otherwise Kruskal–Wallis. The normality of the residuals was evaluated by Shapiro–Wilk test. Post-hoc analysis was performed by Tukey method when the homoscedasticity (homogeneity of the variance) was verified or Games–Howell test in presence of heteroscedasticity. Homoscedasticity was verified by check of the box-plots relatively to three groups and also analyzing the studentized residuals.

Analysis of variables pre and post-purification of each group ((native, ^99m^Tc-polymers (PLGA-NPs) and ^99m^Tc-PVA (PLGA-NPs)) was performed by paired t test and the normality of the differences pre–post was evaluated by Shapiro–Wilk test. Wilcoxon test was used when the normality of the differences failed. 

The stability over time (baseline vs. 1 h vs. 3 h vs. 6 h vs. 24 h) was analyzed by GLIMMIX (Generalized Linear Mixed Model) with Gaussian distribution and identity link for repeated measures. 

The analysis of interaction between the groups (^99m^Tc-PVA (PLGA-NPs) and ^99m^Tc-polymers (PLGA-NPs)) and time (group*time) relative to organs was tested by GLIMMIX (Generalized Linear Mixed Model) with Gaussian distribution and identity link.

The multiple comparisons were corrected by Benjamini–Hochberg (FDR) method. All analyses were performed by SAS v. 9.4 and JMP PRO v. 16 (SAS Institute Inc., Cary, NC, USA). A *p*-value < 0.05 was considered detectable. 

## 4. Results

### 4.1. Particle Size Distribution and Zeta Potential (ζ) Measurements 

**Figure 1:**
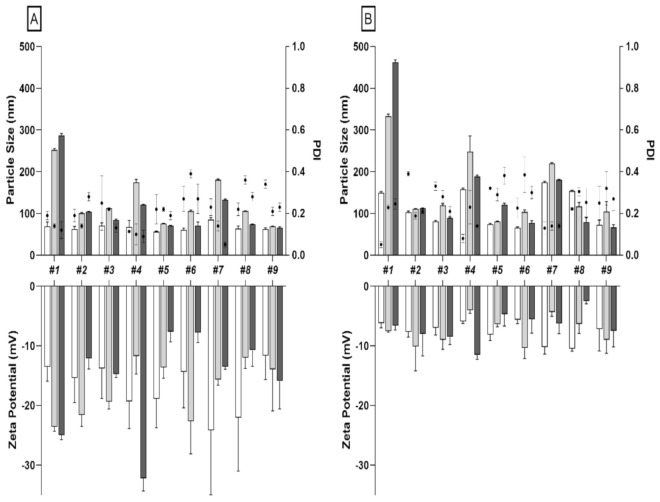
(**A**) Particle size distribution and zeta potential measurements (bars) and PDI (dot) for each formulation of native (white bars), ^99m^Tc-PVA (PLGA-NPs) (grey bars), and ^99m^Tc-polymers (PLGA-NPs) (black bars) pre-PD10 purification. (**B**) Particle size distribution and zeta potential measurements (bars) and PDI (dot) for each formulation of native (white bars), ^99m^Tc-PVA (PLGA-NPs) (grey bars) and ^99m^Tc-polymers (PLGA-NPs) (black bars) post-PD10 purification.

To assess the effects of different microfluidic parameters (batch #1 to #9) on NPs size, charge and PDI, we first synthetized and analyzed native PLGA-NPs. DLS results confirmed that particle size is affected by both TFR and FRR as already published by others [24]. Indeed, by increasing TFR of the two phases (from 8 to 15 mL/min) and by varying FRR from 1:1 to 5:1 (aqueous:organic phase), we detected a reduction in size, especially for radiolabeled NPs (Figure 1, Table S1). The uniformity on NPs size distribution was confirmed by PDI values, ranging from 0.176 to 0.273 (Table S2). All batches had a negative surface charge (ZP) ranging from −11.62 to −24.14 mV (Table S3). A purification step, using PD-10 desalting column, was also performed for native PLGA-NPs to remove the ACN present in solution after synthesis. After PD10 purification we observed a considerable difference in size for all tested batches, except for batch #9 (Table S4).

In particular, the formulation with an FRR of 1:1 and 3:1 at different TFRs (8, 12, or 15 mL/min) showed the greatest increase in size. By contrast, all formulations with FRR of 5:1 and TFR of 8, 12, or 15 mL/min, had a small increase in size. (Figure 1). 

PDI values after purification showed no detectable differences (Table S5).

Zeta potential values of native NPs showed a detectable decrease after purification only for batch #1 (Table S6).

The same formulations of native NPs were used to synthetize radiolabeled NPs. 

Independently from the radiolabeling method (^99m^Tc-PVA- or ^99m^Tc-polymers- PLGA-NPs), all formulations showed detectable increase in size after labeling as compared to the respective native formulation (Figure 1, Table S1). ^99m^Tc-PVA-PLGA-NPs of batch #9 did not show a relivable increase in size after radiolabeling, retaining small size: *p* 63.11 ± 2.53 nm vs. 65.80 ± 2.28 nm, *p* = 0.18 (Table S1).

Similarly to native NPs, labeled NPs showed an increase in size after PD10 purification. In particular, relivable difference was shown as follows: for batches #1, #2, #4, #5, #7, and #9 of ^99m^Tc-polymers (PLGA-NPs) with *p* = 0.009, *p* = 0.006, *p* = 0.03, *p* = 0.006, *p* = 0.003, *p* = 0.01, respectively; for batches #1, #2, #3, #4, #5, #6, and #7 of ^99m^Tc-PVA (PLGA-NPs) with *p* = 0.0007, *p* = 0.008, *p* = 0.02, *p* = 0.0009, *p* = 0.001, *p* = 0.02, *p* = 0.0006, respectively (Figure 1, Table S4). 

The smallest NPs size was obtained by labeling batches #5, #6, #8, and #9 with ^99m^Tc dissolved in aqueous phase (Table S1). 

Zeta potential decreased after purification, with values relivable only for batches #1, #3, #4, #5, and #7 of ^99m^Tc-polymers (PLGA-NPs) (Table S6). 

PDI did not show detectable modifications after PD10 purification for batches #3, #6, #7, and #8 of ^99m^Tc-polymers (PLGA-NPs) and for batches #2, #4, #6, #7, #8, and #9 of ^99m^Tc-PVA (PLGA-NPs) (Table S5). 

### 4.2. “In Vitro” Studies

ITLC results showed a high LE for all batches ranging from 73 to 99% for ^99m^Tc-polymers-(PLGA-NPs) and from 85 to 99% for ^99m^Tc-PVA-(PLGA-NPs), as shown in Table 3. Each batch was then purified with a PD-10 desalting columns to remove unlabeled technetium and other small contaminants, obtaining a final LE of 100%. Radiolabeled yield after purification ranged from 11 to 34% for ^99m^Tc-polymers (PLGA-NPs), and from 19 to 41% for ^99m^Tc-PVA (PLGA-NPs). 

ITLC results showed a high LE of both radiolabeled PLGA-NPs up to 6 h, with a slight decrease at 24 h (Table 4).

**Figure 2:**
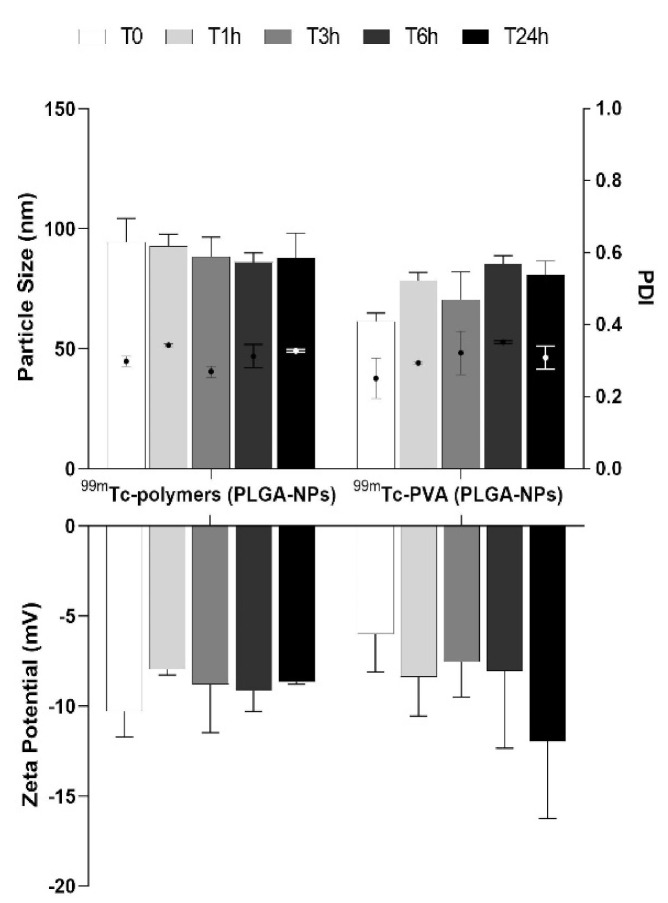
Particle size distribution and zeta potential measurements (bars) and PDI (dot) of batch #9 for both ^99m^Tc-PVA (PLGA-NPs) and ^99m^Tc-polymers (PLGA-NPs) at different time points (1, 3, 6, 24 h).

DLS results showed no significant differences in size and zeta potential over time for both ^99m^Tc-PVA (PLGA-NPs) and ^99m^Tc-polymers (PLGA-NPs) (Figure 2).

### 4.3. In Vivo Biodistribution Studies

Due to their small size, high LE%, and radiochemical stability, ^99m^Tc-PVA (PLGA-NPs) and ^99m^Tc-polymers (PLGA-NPs) from batch #9 were selected for biodistribution studies in BALB/c mice. 

**Figure 3:**
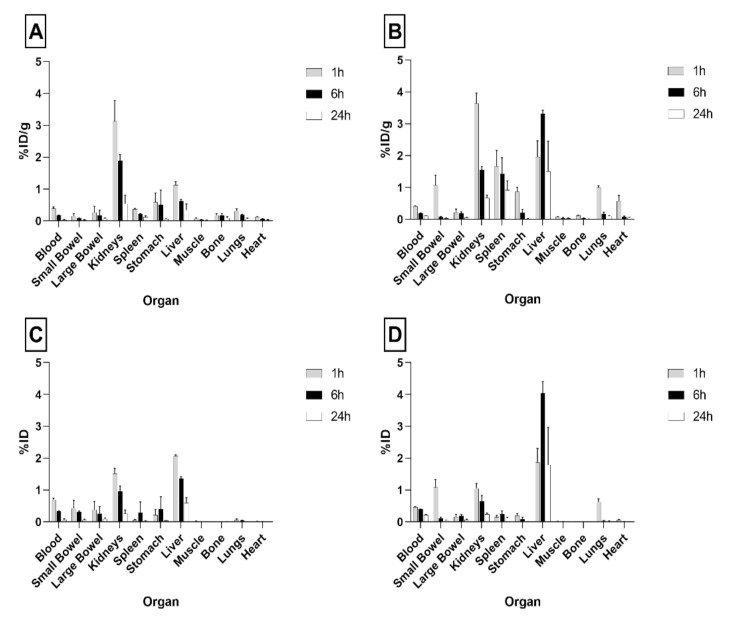
Biodistribution of ^99m^Tc-PVA (PLGA-NPs) (**A**,**C**) and ^99m^Tc-polymers (PLGA-NPs) (**B**,**D**) batch #9 in BALB/c mice. Data are ex vivo counts of each organ at different time points (1, 6, 24 h). The measured activity is expressed as %ID/organ (mean ± SD) (**A**,**B**) and %ID (mean ± SD) (**C**,**D**) detected from three different mice per time point.

As shown in Figure 3, single organ counting at 1 h post-injection (p.i.) indicated a rapid clearance of radiolabeled PLGA-NPs from the bloodstream and their accumulation in liver and kidneys. In particular, at 1 h p.i. the highest uptake was detected in the liver with values of 2.07 ± 0.03 and 1.87 ± 0.43%ID per organ, for ^99m^Tc-PVA (PLGA-NPs) and ^99m^Tc-polymers (PLGA-NPs), respectively. The kidneys showed the highest dose per gram of tissue at 1 h p.i.: 3.13 ± 0.65 and 3.64 ± 0.32%ID/g, for ^99m^Tc-PVA (PLGA-NPs) and ^99m^Tc-polymers (PLGA-NPs), respectively. Nevertheless, kidney kinetics was fast and radioactivity almost disappeared after 24 h for both formulations ((1 h vs. 24 h), *p* < 0.0001 for ^99m^Tc-polymers (PLGA-NPs), and (1 h vs. 24 h) *p* = 0.0005 for ^99m^Tc-PVA (PLGA-NPs)).

Overall, ^99m^Tc-polymers (PLGA-NPs) showed higher uptake in the liver than ^99m^Tc-PVA (PLGA-NPs) with a relevant difference at 6 h ((^99m^Tc-polymers (PLGA-NPs) vs. ^99m^Tc-PVA (PLGA-NPs), %ID/g *p* = 0.006 and %ID per organ *p* = 0.01)) and with significant organ retention up to 24 h (Figure 3).

Tissues or organs such as muscle, bones, large bowel, and heart showed low or negligible uptake of both radiolabeled NPs. 

## 5. Discussion

Physicochemical characteristics of NPs such as size and PDI have an important impact for their in vivo distribution and, therefore, for their application. In the process of NPs synthesis, obtaining reproducible results is a major challenge [25]. The innovative microfluidic technique allows to set different parameters to have dimensional uniformity and low polydispersity of samples [26]. PDI value is important as an indicator of different particles size in solution, that could have different pharmacokinetics profiles in vivo. Values below 0.2 in PDI indicate a high monodisperse solution [27]. 

Zeta potential depends on the composition of the polymer and indicates the stability of the solution. Values close to zero indicate a low repulsion between the NPs which can lead to the formation of colloids [28]. Stolnik et al. reported as zeta potential of PLGA-NPs, in neutral buffer without the presence of PVA in the aqueous phase, a value of about −45 mV, due to the uncapped end carboxyl groups in the particle surface [29]. The use of PVA 2% in aqueous phase, during the synthesis process, reduces the zeta potential of PLGA-NPs. Furthermore, by increasing PVA concentration in solution, the zeta potential becomes less negative, with possible increase of sample instability and NP aggregation over time [30,31]. However, in our experiments, we did not observe NP aggregation as zeta potential remained between −7 and −12 mV. Indeed, PVA being a non-ionic surfactant may promote emulsification of solutions, avoiding aggregation of PLGA-NPs. 

Several studies have demonstrated that the innovative microfluidic technique allows to encapsulate drugs or small-RNA during the synthesis of PLGA-NPs with reproducible results [32]. Chiesa et al. showed that a small hydrophilic drug can be easily encapsulated in PLGA-NPs with the novel microfluidic-based device. They demonstrated that settings of 15 mL/min TFR and a 1:5 FRR, provided the best encapsulation efficiency over 65% [33]. Furthermore, they showed that using the bulk mixing nanoprecipitation method there was a high drug release from NPs (90%) in the first 60 min, compared to 20% of release with microfluidic method. This is important to explain how the microfluidic technique allows a drug or a radioisotope to be encapsulated inside the NPs, avoiding the rapid drug release that occurs when they are absorbed on the surface of NPs. A similar approach was used to encapsulate a hydrophobic drug on PLGA-NPs by Garg et al. They tested different TFR parameters (2–12 mL/min) to reach the best drug encapsulation efficiency. A TFR of 12 mL/min and FRR of 1:1 showed the maximum encapsulation of 75% [34]. 

In our study, we found the formulation parameters of batch #9 (TFR 15 mL/min and FRR 5:1) as the best formulation in terms of LE, labeling yield and size, before and after PD10 purification.

To perform the encapsulation, the drug can be dissolved in the organic or in aqueous phase. For this reason, in our study we explored two different approaches of encapsulation: dissolving the ^99m^TcO_4_^−^ in ACN or in PVA aqueous phase. The advantage of incorporating a radioisotope into NPs is to avoid its direct exposition to biological molecules in vivo, that could lead to radiochemical instability. Furthermore, the encapsulation of ^99m^TcO_4_^−^ allows to avoid the use of reducing agents (as SnCl_2_), and consequently the possible formation of radiocolloids [35]. The low release of ^99m^TcO_4_^−^ from NPs up to 6 h also confirmed the encapsulation of the radioisotope on NPs, avoiding the instability that occurs when it is absorbed on NPs surface.

It has been previously demonstrated that, in vivo, increased NPs size correlates with increased recognition by the mononuclear phagocytic system (MPS), like Kupffer cells in the liver, red-pulp macrophages in the spleen and alveolar macrophages in lungs [36].

Indeed, both formulations analyzed in our in vivo study showed some uptake in liver, spleen, and lungs, as primary organs of the reticuloendothelial system (Figure 3). ^99m^Tc-polymers (PLGA-NPs) with a colloidal size greater than ^99m^Tc-PVA-(PLGA-NPs), showed much higher retention in these organs, particularly in liver (Figure 3A,B).

Of particular note is the kidney uptake of radiolabeled NPs.

Considering that the effective threshold of the glomerulus filtration apparatus for circulating particles is approximately 10 nm, the kidney uptake can be explained by the size of NPs [37]. Indeed, the glomerulus filtration apparatus is composed by three layers with different size threshold. The first barrier is composed by the fenestrated endothelial cells with a size cut-off of ~100 nm. Then, NPs with size below 100 nm are able to access the endothelial fenestration, reaching the mesangium [38]. Choi et al. demonstrated that NPs with a size between 20 and 100 nm passing through the endothelial pores accumulate in the mesangium in different percentages depending on their size. In particular, it has been shown that NPs with size ~50 nm (hydrodynamic size in water ~70 nm) have the highest accumulation in the mesangium due to phagocytosis of mesangial cells [39]. After digestion and degradation ^99m^TcO_4_^−^ is released in the urine with sharp reduction of renal activity.

In our study, both ^99m^Tc-PVA (PLGA-NPs) and ^99m^Tc-polymers (PLGA-NPs) showed some kidney uptake (Figure 3C,D). Nevertheless, ^99m^Tc-polymers (PLGA-NPs) did accumulate much more in other RES tissues such as the liver (Figure 3D).

The low accumulation of radioisotope in the stomach indicated a low release of isotope from NPs, in that free ^99m^TcO_4_^-^ normally accumulated in this organ [40].

More experiments are now being constructed using several formulations of NPs and different tumor models to investigate the role of NP size in tumor targeting.

## 6. Conclusions

In this study, we selected an efficient radiolabeling procedure for PLGA-NPs using an innovative microfluidic method. The ability to load the isotope into PLGA-NPs in a single step, with high yield, allows PLGA-NPs to be considered an attractive candidate for molecular imaging and therapy. Further studies are necessary to explore the tumor binding and retention capacity of these NPs.

## Figures and Tables

**Table 1:** Selected parameters for the formulation of PLGA-NPs using NanoAssemblr^®^. FRR = flow rate ratio; TFR = total flow rate.

Batch	PLGA (mg/mL)	FRR (Aqueous:Organic)	TFR (mL/min)
#1	5	1:1	8
#2	5	3:1	8
#3	5	5:1	8
#4	5	1:1	12
#5	5	3:1	12
#6	5	5:1	12
#7	5	1:1	15
#8	5	3:1	15
#9	5	5:1	15

**Table 2:** Effective incorporation of ^99m^TcO_4_^−^ based on the amount of aqueous and solvent phase used for the synthesis of ^99m^Tc-PLGA-NPs.

FRR (Aqueous:Organic)	PVA (mL)	PLGA Polymers (mL)	^99m^TcO_4_^−^ in PVA (mCi/mL)	^99m^TcO_4_^−^ in PLGA Polymers (mCi/mL)
1:1	1	1	5	-
3:1	1.5	0.5	3.3	-
5:1	1.67	0.33	2.99	-
1:1	1	1	-	5
3:1	1.5	0.5	-	10
5:1	1.67	0.33	-	15.15

**Table 3:** Encapsulation efficiency (%) and yield (%) of ^99m^Tc-PVA (PLGA-NPs) and ^99m^Tc-polymers (PLGA-NPs). Results represent triplicate measurements.

Batch	^99m^Tc-Polymers (PLGA-NPs)		^99m^Tc-PVA (PLGA-NPs)	
N°	LE Mean ± SD	YIELD Mean ± SD	LE Mean ± SD	YIELD Mean ± SD
#1	96.87 ± 1.74	26.70 ± 0.14	98.25 ± 2.47	19.06 ± 0.72
#2	99.25 ± 1.06	34.05 ± 0.19	99.20 ± 1.13	27.50 ± 0.58
#3	98.83 ± 0.40	22.54 ± 0.26	99.45 ± 0.78	31.60 ± 0.36
#4	95.50 ± 1.20	22.12 ± 0.17	99.61 ± 0.56	32.93 ± 0.32
#5	85.09 ± 10.96	11.60 ± 0.88	85.28 ± 17.23	29.82 ± 0.18
#6	73.15 ± 26.90	14.78 ± 0.17	92.57 ± 10.51	41.33 ± 0.07
#7	95.70 ± 1.48	19.10 ± 0.21	95.10 ± 2.06	31.73 ± 0.21
#8	95.11 ± 1.92	13.8 ± 0.10	85.83 ± 5.25	20.71 ± 0.27
#9	88.53 ± 1.43	10.92 ± 0.15	94.20 ± 5.83	33.69 ± 0.22

**Table 4:** Labeling efficiency calculation (%) at different time points (1, 3, 6, and 24 h) to evaluate the technetium-99m release in 0.9% NaCl from ^99m^Tc-PVA (PLGA-NPs) and ^99m^Tc-polymers (PLGA-NPs) batch #9. Results represent triplicate measurements.

^99mTc^PLGA-NPs	1 h Mean ± SD	3 h Mean ± SD	6 h Mean ± SD	24 h Mean ± SD
99mTc-polymers	98.34 ± 1.79	97.97 ± 1.82	97.92 ± 1.86	84.34 ± 7.57
99mTc-PVA	97.17 ± 3.66	97.92 ± 2.38	97.13 ± 2.65	84.21 ± 8.22

## Data Availability

The data presented in this study are available within this article. Any other data is available on request from the corresponding author.

## Supplementary Materials

The following are available online at https://www.mdpi.com/article/10.3390/pharmaceutics13111769/s1, Figure S1: Schematic illustration of two different radiolabeling approaches during ^99m^Tc-NPs synthesis via microfluidic technique method using a Dean-Vortex-Bifurcation Mixer (DVBM) cartridge, Table S1: Particle size distribution (nm) of native-, ^99m^Tc-PVA-, and ^99m^Tc-polymers -(PLGA-NPs) pre-PD10 purification, Table S2: PDI values of native-, ^99m^Tc-PVA-, and ^99m^Tc-polymers -(PLGA-NPs) pre-PD10 purification, Table S3: Zeta potential measurements (mV) of native-, ^99m^Tc-PVA-, and ^99m^Tc-polymers -(PLGA-NPs) pre-PD10 purification, Table S4: Particle size distribution (nm) of native-, ^99m^Tc-PVA-, and ^99m^Tc-polymers -(PLGA-NPs) post-PD10 purification, Table S5: PDI values of native-, ^99m^Tc-PVA-, and ^99m^Tc-polymers -(PLGA-NPs) post-PD10 purification, Table S6: Zeta potential measurements (mV) of native-, ^99m^Tc-PVA-, and ^99m^Tc-polymers (PLGA-NPs) post-PD10 purification.
