# Supplementary material for: Synthesis and Biodistribution of 99mTc-Labeled PLGA Nanoparticles by Microfluidic Technique
Source: Pharmaceutics. 2021 Oct 22;13(11):1769. doi: 10.3390/pharmaceutics13111769 (PMC8621482; doi:10.3390/pharmaceutics13111769)
Supplement: Supplementary file 1 [file pharmaceutics-13-01769-s001.zip › pharmaceutics-1426038-supplementary.pdf]

pharmaceutics

Supplementary Materials: Synthesis and biodistribution of ^{99m}Tc-labelled PLGA nanoparticles by microfluidic technique

Michela Varani, Giuseppe Campagna, Valeria Bentivoglio, Matteo Serafinelli, Maria Luisa Martini, Filippo Galli and Alberto Signore

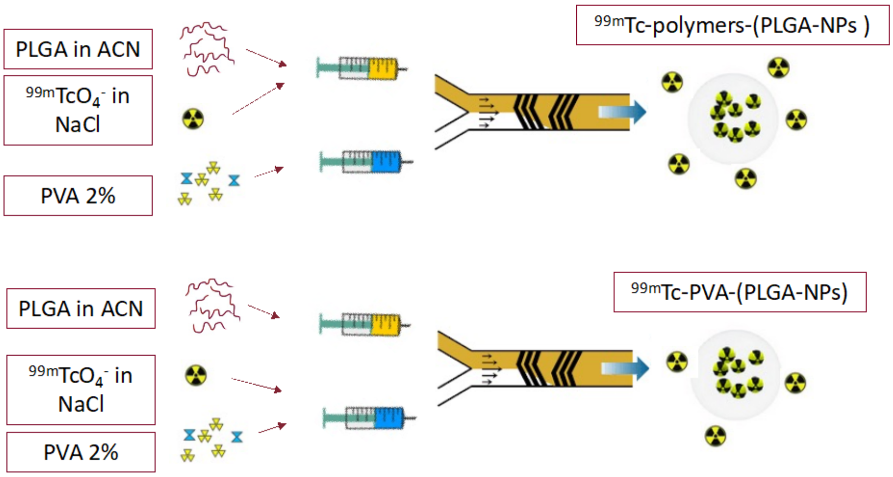

Figure S1. Schematic illustration of two different radiolabelling approaches during ^{99m}Tc-NPs synthesis via microfluidic technique method using a Dean-Vortex-Bifurcation Mixer (DVBM) cartridge. PLGA: poly(lactic-co-glycolic acid); ACN: acetonitrile; PVA: polyvinyl alcohol.

Table S1. Particle size distribution (nm) of native-, ^{99m}Tc-PVA- and ^{99m}Tc-polymers -(PLGA-NPs) pre-PD10 purification. A p-value < 0.05 was considered detectable.

Batch	Native Mean ± SD (95%CI)	^{99m}Tc-polymers Mean ± SD (95%CI)	^{99m}Tc-(PVA) Mean ± SD (95%CI)	Native vs. ^{99m}Tc-polymers *p*	^{99m}Tc-polymers vs. ^{99m}Tc-(PVA) *p*	Native vs. ^{99m}Tc-(PVA) *p*
#1	69.46 ± 10.2 (58.76 to 80.16)	252.63 ± 3.07 (245.02 to 260.25)	287.47 ± 4.16 (277.12 to 297.81)	**< 0.0001**	**< 0.0001**	**0.001**
#2	62.05 ± 6.90 (54.81 to 69.29)	100.36 ± 1.94 (95.54 to 105.18)	104.13 ± 0.76 (102.24 to 106.03)	**< 0.0001**	**< 0.0001**	0.12
#3	71.05 ± 6.42 (64.31 to 77.79)	112.33 ± 0.76 (110.44 to 114.23)	84.71 ± 1.47 (81.07 to 88.35)	**< 0.0001**	**0.0002**	**0.006**
#4	68.13 ± 14.97 (52.42 to 83.84)	174.43 ± 7.03 (167.05 to 181.81)	121.33 ± 0.60 (119.84 to 122.83)	**< 0.0001**	**0.0008**	**< 0.0001**
#5	56.67 ± 0.93 (55.69 to 57.65)	75.32 ± 1.24 (72.25 to 78.39)	70.22 ± 1.74 (68.39 to 72.05)	**< 0.0001**	**0.0005**	**< 0.0001**
#6	60.02 ± 5.19 (54.58 to 65.46)	105.60 ± 2.45 (99.51 to 111.69)	71.28 ± 8.18 (62.69 to 79.86)	**< 0.0001**	**0.0001**	**0.048**
#7	85.27 ± 5.02 (80.01 to 90.54)	180.20 ± 2.46 (174.10 to 186.3)	132.80 ± 1.56 (128.92 to 136.68)	**< 0.0001**	**< 0.0001**	**< 0.0001**
#8	63.44 ± 6.28 (56.84 to 70.03)	105.63 ± 0.55 (104.27 to 107.00)	74.12 ± 1.09 (71.41 to 76.84)	**< 0.0001**	**< 0.0001**	**0.02**
#9	63.11 ± 2.53 (60.46 to 65.77)	68.90 ± 1.34 (67.49 to 70.30)	65.80 ± 2.28 (63.41 to 68.20)	**0.003**	**0.049**	0.18

Table S2. PDI values of native-, ^{99m}Tc-PVA- and ^{99m}Tc-polymers -(PLGA-NPs) pre-PD10 purification. A p-value < 0.05 was considered detectable.

Batch	Native mean±SD (95%CI)	^{99m}Tc-polymers mean±SD (95%CI)	^{99m}Tc-(PVA) mean±SD (95%CI)	Native vs. ^{99m}Tc-polymers *p*	^{99m}Tc-polymers vs. ^{99m}Tc-(PVA) *p*	Native vs. ^{99m}Tc-(PVA) *p*
#1	0.19 ± 0.02 (0.17 to 0.21)	0.14 ± 0.01 (0.13 to 0.15)	0.12 ± 0.04 (0.02 to 0.21)	**0.003**	0.60	0.12
#2	0.19 ± 0.03 (0.16 to 0.23)	0.14 ± 0.01 (0.11 to 0.16)	0.28 ± 0.02 (0.23 to 0.34)	**< 0.0001**	**< 0.0001**	**0.0008**
#3	0.25 ± 0.13 (0.11 to 0.39)	0.22 ± 0.00 (0.21 to 0.23)	0.13 ± 0.02 (0.08 to 0.18)	0.91	0.25	0.53
#4	0.14 ± 0.03 (0.11 to 0.17)	0.1 ± 0.05 (0.04 to 0.15)	0.09 ± 0.03 (0.02 to 0.15)	0.21	0.95	0.22
#5	0.22 ± 0.07 (0.15 to 0.29)	0.22 ± 0.01 (0.18 to 0.25)	0.19 ± 0.02 (0.17 to 0.21)	1.00	0.70	0.59
#6	0.27 ± 0.06 (0.21 to 0.34)	0.39 ± 0.02 (0.34 to 0.44)	0.27 ± 0.07 (0.19 to 0.35)	**0.01**	**0.02**	1.00
#7	0.23 ± 0.04 (0.19 to 0.27)	0.14 ± 0.02 (0.09 to 0.2)	0.05 ± 0.01 (0.02 to 0.08)	**0.01**	**0.02**	**< 0.0001**
#8	0.22 ± 0.03 (0.19 to 0.26)	0.36 ± 0.02 (0.32 to 0.40)	0.28 ± 0.02 (0.23 to 0.33)	**< 0.0001**	**0.009**	**0.03**
#9	0.34 ± 0.02 (0.32 to 0.36)	0.21 ± 0.02 (0.18 to 0.23)	0.23 ± 0.02 (0.21 to 0.25)	**< 0.0001**	0.15	**< 0.0001**

Table S3. Zeta potential measurements (mV) of native-, ^{99m}Tc-PVA- and ^{99m}Tc-polymers -(PLGA-NPs) pre-PD10 purification. A p-value < 0.05 was considered detectable.

Batch	Native mean±SD (95%CI)	^{99m}Tc-polymers mean±SD (95%CI)	^{99m}Tc-(PVA) mean±SD (95%CI)	Native vs. ^{99m}Tc-polymers *p*	^{99m}Tc-polymers vs. ^{99m}Tc-(PVA) *p*	Native vs. ^{99m}Tc-(PVA) *p*
#1	−13.55 ± 2.37 (−16.04 to −11.06)	−23.57 ± 0.76 (−25.46 to −21.67)	−24.97 ± 0.78 (−26.90 to −23.04)	**0.0001**	0.18	**< 0.0001**
#2	−15.40 ± 4.15 (−19.75 to −11.04)	−21.60 ± 1.91 (−26.34 to −16.86)	−12.13 ± 1.75 (−16.47 to −7.79)	**0.04**	**0.007**	0.29
#3	−13.80 ± 5.04 (−19.09 to −8.50)	−19.37 ± 1.23 (−22.43 to −16.30)	−14.73 ± 0.60 (−16.23 to −13.24)	0.15	0.34	0.94
#4	−19.30 ± 4.58 (−24.10 to −14.50)	−11.73 ± 2.97 (−14.84 to −8.61)	−32.20 ± 2.15 (−37.55 to −26.85)	**0.02**	**< 0.0001**	**0.002**
#5	−18.87 ± 4.90 (−24.01 to −13.72)	−13.60 ± 1.84 (−18.16 to −9.04)	−7.61 ± 1.73 (−9.42 to −5.79)	0.12	**0.02**	**0.004**
#6	−14.40 ± 6.02 (−20.72 to −8.08)	−22.63 ± 5.50 (−36.30 to −8.97)	−7.77 ± 1.69 (−9.54 to −6.00)	0.06	**0.002**	0.07
#7	−24.14 ± 13.02 (−37.80 to −10.47)	−15.67 ± 0.95 (−18.01 to −13.32)	−13.5 ± 0.44 (−14.58 to −12.42)	0.46	0.96	0.32
#8	−22.03 ± 8.97 (−31.44 to −12.62)	−11.97 ± 1.79 (−16.41 to −7.52)	−10.75 ± 2.71 (−17.47 to −4.02)	0.15	0.97	0.11
#9	−11.62 ± 4.02 (−15.83 to −7.40)	−13.93 ± 6.96 (−21.23 to −6.63)	−15.90 ± 4.71 (−20.84 to −10.96)	0.74	0.81	0.38

Table S4. Particle size distribution (nm) of native-, ^{99m}Tc-PVA- and ^{99m}Tc-polymers -(PLGA-NPs) post-PD10 purification. A p-value < 0.05 was considered detectable.

Batch	Native mean±SD (95%CI)	^{99m}Tc-polymers mean±SD (95%CI)	^{99m}Tc-(PVA) mean±SD (95%CI)	Native vs. ^{99m}Tc-polymers *p*	^{99m}Tc-polymers vs. ^{99m}Tc-(PVA) *p*	Native vs. ^{99m}Tc-(PVA) *p*
#1	149.83 ± 2.65 (143.25 to 156.42)	332.67 ± 5.47 (319.08 to 346.25)	461.60 ± 6.62 (445.16 to 478.04)	**< 0.0001**	**< 0.0001**	**0.001**
#2	103.03 ± 2.96 (95.69 to 110.38)	110.97 ± 1.33 (107.67 to 114.27)	112.57 ± 1.03 (110.02 to 115.12)	0.055	0.33	**0.04**
#3	80.55 ± 2.49 (74.36 to 86.74)	119.60 ± 3.25 (111.52 to 127.68)	88.69 ± 2.31 (82.95 to 94.43)	**< 0.0001**	**< 0.0001**	**0.02**
#4	158.37 ± 2.33 (152.58 to 164.15)	248.75 ± 36.99 (209.93 to 287.57)	188.57 ± 2.90 (181.37 to 195.77)	**0.004**	**0.02**	**0.0004**
#5	74.11 ± 2.62 (67.61 to 80.61)	80.56 ± 1.50 (76.83 to 84.29)	120.97 ± 3.46 (112.37 to 129.56)	0.06	**< 0.0001**	**< 0.0001**
#6	65.09 ± 2.80 (58.14 to 72.04)	103.54 ± 5.21 (90.59 to 116.49)	77.57 ± 5.04 (72.28 to 82.86)	**< 0.0001**	**< 0.0001**	**0.01**
#7	174.93 ± 2.55 (168.60 to 181.27)	219.53 ± 2.28 (213.87 to 225.20)	180.53 ± 1.40 (177.04 to 184.02)	**< 0.0001**	**< 0.0001**	**0.04**
#8	153.87 ± 1.76 (149.49 to 158.24)	117.03 ± 9.50 (93.42 to 140.64)	79.08 ± 10.61 (52.71 to 105.45)	**0.03**	**0.02**	**0.01**
#9	73.20 ± 10.88 (46.17 to 100.23)	104.69 ± 23.59 (79.93 to 129.44)	66.62 ± 6.71 (59.57 to 73.66)	0.07	**0.02**	0.65

Table S5. PDI values of native-, ^{99m}Tc-PVA- and ^{99m}Tc-polymers -(PLGA-NPs) post-PD10 purification. A p-value < 0.05 was considered detectable.

Batch	Native mean±SD (95%CI)	^{99m}Tc-polymers mean±SD (95%CI)	^{99m}Tc-(PVA) mean±SD (95%CI)	Native vs. ^{99m}Tc-polymers *p*	^{99m}Tc-polymers vs. ^{99m}Tc-(PVA) *p*	Native vs. ^{99m}Tc-(PVA) *p*
#1	0.05 ± 0.01 (0.01 to 0.09)	0.23 ± 0.01 (0.22 to 0.24)	0.25 ± 0.02 (0.18 to 0.31)	**0.002**	0.58	**0.002**
#2	0.39 ± 0.01 (0.36 to 0.42)	0.19 ± 0.01 (0.16 to 0.22)	0.21 ± 0.01 (0.19 to 0.23)	**< 0.0001**	0.11	**< 0.0001**
#3	0.33 ± 0.02 (0.28 to 0.38)	0.28 ± 0.03 (0.20 to 0.35)	0.21 ± 0.02 (0.15 to 0.26)	0.07	**0.03**	**00002**
#4	0.08 ± 0.02 (0.03 to 0.13)	0.23 ± 0.08 (0.14 to 0.32)	0.14 ± 0.00 (0.13 to 0.14)	**0.014**	0.09	0.06
#5	0.32 ± 0.00 (0.32 to 0.33)	0.29 ± 0.03 (0.22 to 0.35)	0.38 ± 0.04 (0.29 to 0.47)	0.28	**0.009**	0.06
#6	0.27 ± 0.05 (0.15 to 0.38)	0.38 ± 0.08 (0.17 to 0.60)	0.30 ± 0.03 (0.28 to 0.33)	**0.04**	0.11	0.54
#7	0.13 ± 0.03 (0.05 to 0.20)	0.14 ± 0.02 (0.10 to 0.18)	0.14 ± 0.01 (0.11 to 0.16)	0.69	0.99	0.73
#8	0.22 ± 0.00 (0.21 to 0.23)	0.30 ± 0.02 (0.26 to 0.35)	0.25 ± 0.07 (0.08 to 0.42)	0.11	0.34	0.65
#9	0.25 ± 0.08 (0.04 to 0.45)	0.32 ± 0.08 (0.24 to 0.40)	0.27 ± 0.05 (0.22 to 0.32)	0.35	0.49	0.88

Table S6. Zeta potential measurements (mV) of native-, ^{99m}Tc-PVA- and ^{99m}Tc-polymers -(PLGA-NPs) post-PD10 purification. A p-value < 0.05 was considered detectable.

Batch	Native mean±SD (95%CI)	^{99m}Tc-polymers mean±SD (95%CI)	^{99m}Tc-(PVA) mean±SD (95%CI)	Native vs. ^{99m}Tc-polymers *p*	^{99m}Tc-polymers vs. ^{99m}Tc-(PVA) *p*	Native vs. ^{99m}Tc-(PVA) *p*
#1	−6.16 ±0.81 (-8.17 to -4.15)	−7.54 ± 0.17 (−7.96 to −7.11)	−6.63 ± 0.74 (−8.48 to −4.78)	0.09	0.27	0066
#2	−7.67 ±0.88 (−9.86 to −5.48)	−10.14 ± 4.10 (−20.32 to 0.04)	−7.97 ± 3.74 (−17.26 to 1.32)	0.64	0071	0.99
#3	−6.99 ±1.22 (−10.02 to −3.95)	−9.00 ± 1.59 (−12.94 to −5.06)	−8.53 ± 1.25 (−11.63 to −5.43)	0.24	0.91	0.41
#4	−5.85 ±0.39 (−6.81 to −4.90)	−3.99 ± 0.56 (−4.57 to −3.40)	−11.47 ± 0.75 (−13.33 to −9.60)	**0.003**	**< 0.0001**	**< 0.0001**

#5	−8.12 ± 1.00 (−10.60 to −5.65)	−6.34 ± 0.47 (−7.51 to −5.16)	−4.66 ± 2.05 (−9.76 to 0.44)	0.31	0.34	0.052
#6	−5.58 ± 0.70 (−7.32 to −3.84)	−10.42 ± 1.72 (−14.70 to −6.14)	−5.49 ± 2.39 (−8.00 to −2.98)	0.052	**0.03**	0.99
#7	−10.21 ± 1.18 (−13.13 to −7.29)	−4.32 ± 0.73 (−6.13 to −2.50)	−6.27 ± 1.69 (−10.46 to −2.08)	**0.003**	0.22	**0.02**
#8	−10.53 ± 0.40 (−11.54 to −9.53)	−6.31 ± 1.61 (−10.31 to −2.32)	−2.47 ± 0.48 (−3.65 to −1.29)	0.07	0.08	**< 0.0001**
#9	−7.19 ± 3.68 (−16.33 to 1.95)	−8.99 ± 2.26 (−11.36 to −6.62)	−7.50 ± 2.68 (−10.32 to −4.68)	0.63	0.62	0.99
